# The effect of personal medical history and family history of cancer on the uptake of risk-reducing salpingo-oophorectomy

**DOI:** 10.1007/s10689-015-9827-7

**Published:** 2015-08-12

**Authors:** Jessica E. van der Aa, Jacob P. Hoogendam, Els S. F. Butter, Margreet G. E. M. Ausems, René H. M. Verheijen, Ronald P. Zweemer

**Affiliations:** Department of Gynaecological Oncology, UMC Utrecht Cancer Center, Heidelberglaan 100, 3584 CX Utrecht, The Netherlands; Department of Medical Genetics, University Medical Center Utrecht, Heidelberglaan 100, 3584 CX Utrecht, The Netherlands

**Keywords:** Hereditary ovarian cancer, BRCA1, BRCA2, Risk-reducing salpingo-oophorectomy, Prophylactic surgery uptake

## Abstract

Women with an increased lifetime risk of ovarian cancer are advised to undergo risk-reducing salpingo-oophorectomy (RRSO) to reduce risk of adnexal cancer. We investigated the uptake of RRSO and evaluated the influence of personal medical history of (breast) cancer, risk-reducing mastectomy (RRM) and family history of ovarian and/or breast cancer on the RRSO decision. This single center retrospective observational cohort study was performed in a tertiary multidisciplinary clinic for hereditary cancer of the University Medical Centre Utrecht, the Netherlands. Women ≥35 years old with an estimated lifetime risk of ovarian cancer ≥10 %, who had completed childbearing, were eligible for RRSO. Uptake and timing of RRSO were analyzed. Influence of personal medical history and family history on RRSO decision making, were evaluated with logistic regression. The study population consisted of 218 women (45.0 % BRCA1 mutation carrier, 28.0 % BRCA2 mutation carrier, 27.0 % with familial susceptibility) with 87.2 % RRSO uptake. The median age at RRSO was 44.5 (range 28–73) years. Of the women undergoing RRSO, 78.3 % needed ≤3 consultations to reach this decision. Multivariable analysis showed a significant difference in RRSO uptake for women with a history of RRM [OR 3.66 95 % CI (1.12–11.98)], but no significant difference in women with a history of breast cancer [OR 1.38 95 % CI (0.50–3.79)], nor with a family history of ovarian and/or breast cancer [OR 1.10 95 % CI (0.44–2.76)]. We conclude that RRSO counseling, without the alternative of screening, is effective. The uptake is increased in women with a history of RRM.

## Introduction

Women carrying a BRCA1 or BRCA2 gene mutation have a life time risk of developing ovarian cancer of 31–58.9 and 6–34.5 % respectively [1–5]. Risk-reducing salpingo-oophorectomy (RRSO) has been associated with a risk reduction of 85–96 % for ovarian, fallopian tube and peritoneal cancer [6–9]. A meta-analysis by Marchetti et al. (2014), showed a hazard ratio of 0.19 (95 % CI 0.13–0.27) for the development of ovarian cancer in BRCA1/2 mutation carriers up to six years after RRSO [10]. A large meta-analysis by Rebbeck et al. [11] showed a hazard ratio of 0.21 (95 % CI 0.12–0.39) for the development of ovarian or breast cancer, up to five years after RRSO. Risk reduction of breast cancer after RRSO was estimated to be 53–72 % in BRCA1/2 mutation carriers in several studies [6–9]. A recent Dutch cohort study found no evidence for a risk reduction of breast cancer risk in BRCA1/2 mutation carriers after RRSO [hazard ratio 1.09 (95 % CI 0.67–1.77)] and stated that previous data may have overestimated the risk reduction because of bias [12].

Screening for ovarian cancer by determining CA125 protein serum levels and performing transvaginal ultrasonography, has proven to be neither reliable, nor beneficial in reducing the risk of ovarian cancer, nor does it improve survival [13–19]. Therefore, RRSO is the only scientifically proven strategy to reduce the risk of developing ovarian and fallopian tube cancer and it is recommended to women with an increased lifetime risk [10, 11].

The Multidisciplinary Hereditary Cancer Clinic (MHCC) of the UMC Utrecht aims to counsel and treat women at increased lifetime risk of developing ovarian cancer. This encompasses BRCA1/2 mutation carriers and women with familial susceptibility to ovarian cancer, which is determined by having a pedigree-based estimated lifetime risk of ≥10 % [20]. When over 35 years of age and having completed childbearing, these women become eligible for RRSO [21]. During RRSO counseling, the surgical risks as well as the benefits and indications for Hormonal Replacement Therapy (HRT) after surgery, are outlined. Screening is explicitly not offered as an alternative to RRSO. Psychosocial support is routinely offered in the process of decision making.

Reported RRSO uptake in the literature varies from 10.9 to 75 % [22–34]. With our approach, we expect a high RRSO uptake, since undergoing the surgery is the only risk-reducing strategy.

There are demographic, medical and psychosocial factors that affect the decision making to undergo risk-reducing surgery, including age, parity, menopausal status, ethnicity, education level, idea of perceived risk, level of anxiety or distress, etcetera [22, 23, 25–28, 30, 32–35]. Since BRCA1/2 mutation status and familial susceptibility to ovarian cancer are familial conditions that concern close relatives, it is assumed that decision making may be influenced by personal experience and family perspectives or experience with cancer within the family. Several studies support this by showing an increase of RRSO uptake with a more positive family history of breast and/or ovarian cancer [23, 27, 29, 33]. However, others do not report this [28].

Here, we explore the effect of a personal medical history of breast cancer and risk-reducing mastectomy (RRM) as well as family history of ovarian and/or breast cancer in the decision making of undergoing RRSO for women with an increased lifetime risk of ovarian cancer. Secondly, we determined how many consultations were needed for women to decide to undergo RRSO. This insight is of importance, to provide personally appropriate counseling to every patient.

## Methods

### MHCC and counseling

In this single center retrospective cohort study, we identified all women who visited the MHCC from January 2011 through June 2013. Genetic testing and pedigree-based risk assessment were performed by the medical genetics department. Carriers of a BRCA1 mutation are explained to have a lifetime risk of ovarian cancer of 30–60 % and BRCA2 mutation carriers of 5–20 %. RRSO is recommended from age 35 years onwards (BRCA1 carrier) or from 40 years onwards (BRCA2 carrier). Women with a positive family history, but in whom genetic testing in an affected relative could not be performed, or could not identify a BRCA mutation (e.g. due to uninformative test result), were defined as women with a familial susceptibility to ovarian cancer. They are advised to undergo RRSO when the estimated lifetime risk is ≥10 %.

In adherence to the Dutch guideline for treatment of hereditary ovarian cancer, women were considered eligible for RRSO with an estimated lifetime risk ≥10 % and if they were ≥35 years of age and had completed childbearing [18]. They received counseling for RRSO from a gynecologic oncologist and a nurse specialized in familial cancer care. Counseling was aimed at informing patients about the risks of the surgery, the induction of menopausal status by the surgery and indications for HRT, the medical and psychosocial support available, the risks of developing ovarian and fallopian tube cancer and the reduction of this risk by undergoing RRSO.

### Study population

The study population consists of all women who received counseling for RRSO, based on the criteria described previously. Women who had completed childbearing at <35 years of age and had received counseling for RRSO were included too.

Women previously diagnosed with ovarian cancer, carriers of a mutation in one of the MMR genes (predisposing to Lynch syndrome), women with increased risk of breast cancer or other non-breast and ovarian related hereditary tumors only, were excluded.

### Statistical analysis

Statistical analyses were carried out using the ‘Statistical Package for the Social Sciences’ version 21.0 (SPSS, International Business Machines, New York, United States). Comparison of categorical variables was performed by Chi square testing. The Mann–Whitney *U* test was used for count data and continuous variables that were not normally distributed. A *p* value of <0.05 was considered statistically significant.

Uni- and multivariable logistic regression, producing odds ratios (OR), were used to model the explanatory variables against the binary RRSO decision. Explanatory variables of interest included the effect of personal medical history of breast cancer, any malignancy (excluding all non-melanoma skin malignancies) and RRM. Furthermore, family history of ovarian and/or breast cancer were analyzed according to the first or second/third degree in which they occurred. Multiple imputations (m = 10) were used to correct randomly missing values.

During the multivariable analysis, explanatory variables were adjusted for mutation status, age, parity and menopausal status, since these have been identified as significant factors of influence in previous studies [22, 23, 27–30, 32–34].

## Results

### Study population

We identified 218 women who were considered eligible for undergoing RRSO. As shown in Table 1, the majority carried a BRCA1 or BRCA2 mutation (72.9 %).

**Table 1:** Baseline characteristics of study-population n = 218

Mutation status, n (%)	
BRCA1	98 (45.0 %)
BRCA2	61 (28.0 %)
Familial susceptibility	59 (27.0 %)
Age at first presentation	
Median (range), years	43 (27–77)
Mean (Standard deviation), years	44.63 (±9.37)
Menopausal status at first presentation	
Premenopausal, n (%)	126 (57.8 %)
Postmenopausal, n (%)	70 (32.1 %)
Unknown, n (%)	22 (10.1 %)
Parity, n (%)	
0, n (%)	40 (18.3 %)
1, n (%)	22 (10.1 %)
2, n (%)	88 (40.4 %)
>2, n (%)	46 (21.1 %)
Unknown, n (%)	22 (10.1 %)
Medical history	
No malignancy, n (%)	143 (65.6 %)
Breast cancer, n (%)	70 (32.1 %)
Other malignancy, n (%)	5 (2.3 %)
Family history	
Positive for ovarian cancer, n (%)	102 (46.8 %)
Positive for breast cancer, n (%)	140 (64.2 %)
Positive for breast *and* ovarian cancer, n (%)	76 (34.9 %)
Unknown, n (%)	51 (23.4 %)
RRSO^a^ uptake	
Yes, n (%)	190 (87.2 %)
No, n (%)	28 (12.8 %)
Age at RRSO^a^ (n = 186)	
Median (range), years	44.5 (28–73)
Mean (standard deviation), years	46.32 (±8.24)
RRM^b^ uptake	
Yes, n (%)	107 (49.1 %)
No, n (%)	93 (42.7 %)
Missing, n (%)	18 (8.3 %)

^a^Risk-reducing salpingo-oophorectomy

^b^Risk-reducing mastectomy

In total, 190 women opted for RRSO, resulting in an uptake of 87.2 %. Uptake was higher in BRCA1/2 mutation carriers than in women with familial susceptibility to ovarian cancer (90.6 and 78.0 % respectively).

Median age at RRSO was 44.5 (range 28–73). Median ages at RRSO for BRCA1 mutation carriers, BRCA2 mutation carriers and women with familial susceptibility were 42 (range 30–63), 47 (range 28–73) and 48 years (range 31–65) respectively.

### Number of consultations

We registered the number of consultations women had had with the gynecologic oncologist before they decided to undergo RRSO. It showed that 57.4 % of the women who eventually underwent RRSO, had decided so at the first consultation with the gynecologic oncologist or the specialized nurse. An additional 11.1 % had decided so by the second consultation and an additional 5.3 % had made this decision by the third consultation. The remaining 26.2 % needed four or more consultations to make the decision.

Women with 1st degree relatives who had breast cancer needed significantly less consultations to decide for RRSO, this in contrast to having 1st degree relatives with ovarian cancer. See Table 2 for the univariable analysis. After multivariable adjustment, only a 1st degree relative with breast cancer remained as a significant factor (*p* 0.01) with 1.51 (95 % CI 0.32–2.70) less consultations before the RRSO decision.

**Table 2:** Number of consultations needed to decide for RRSO relative to personal and family history (n = 190)

	Mean ± standard deviation number of consultations^a^		*p* value^b^
	Absent	Present	
Breast cancer in personal medical history	2.29 ± 3.19	2.90 ± 3.02	0.51
Any malignancy in personal medical history^c^	2.35 ± 3.24	2.73 ± 2.93	0.67
RRM^d^ in personal medical history	2.11 ± 2.54	3.10 ± 3.90	0.38
Ovarian cancer in 1st degree relative	2.19 ± 2.80	2.96 ± 3.61	**0.02**
Ovarian cancer in 2nd or 3rd degree relative	2.30 ± 2.90	3.14 ± 3.88	0.33
Breast cancer in 1st degree relative	3.09 ± 3.82	1.90 ± 2.20	**<0.01**
Breast cancer in 2nd or 3rd degree relative	2.19 ± 2.46	3.02 ± 4.11	0.06

Statistically significant *p* values are presented in bold

^a^While ‘number of consultations’ follows a Poisson distribution, results are deliberately presented in means and standard deviations whereas medians and ranges (in integer values) would obscure the subtle differences between groups

^b^Based on the non-parametric Mann–Whitney *U* test

^c^Excludes all non-melanoma skin malignancies

^d^Risk-reducing mastectomy

### Personal and medical history

Table 3 shows the outcomes of univariable and multivariable analyses of RRSO uptake in women with family history of ovarian and/or breast cancer or a personal history of (breast) cancer.

**Table 3:** Personal and family history and its effect on uptake of risk-reducing salpingo-oophorectomy

	RRSO^a^ uptake (%)	Univariable OR^b^	95 % CI^c^	Multivariable^d^ OR^b^	95 % CI^c^
Breast cancer in personal medical history	91.4	1.86	0.72–4.82	1.38	0.50–3.79
Any malignancy in personal medical history^e^	92.0	2.09	0.81–5.41	1.60	0.59–4.37
RRM^f^ in personal medical history	94.7	**3.59**	**1.20–10.73**	**3.66**	**1.12–11.98**
Ovarian cancer in 1st degree relative	84.3	0.52	0.21–1.27	0.61	0.23–1.62
Ovarian cancer in 2nd or 3rd degree relative	90.0	1.27	0.40–4.05	1.30	0.37–4.57
Breast cancer in 1st degree relative	88.8	1.16	0.46–2.91	0.85	0.31–2.33
Breast cancer in 2nd or 3rd degree relative	88.3	1.13	0.46–2.80	1.30	0.49–3.44
Ovarian *and* breast cancer in 1st, 2nd or 3rd degree relative	86.8	1.02	0.42–2.47	1.10	0.44–2.76

Statistically significant odds ratios are presented in bold

^a^Risk-reducing salpingo-oophorectomy

^b^Odds ratio

^c^Confidence interval

^d^Adjusted for mutation status, age, parity and menopausal status

^e^Excludes all non-melanoma skin malignancies

^f^Risk-reducing mastectomy

Women who had previously been diagnosed with breast cancer or any other type of cancer (excluding all non-melanoma skin malignancies), did not have significantly increased odds of undergoing a RRSO [OR 1.38; 95 % confidence interval (CI) (0.50–3.79) and OR 1.60; 95 % CI (0.59–4.37) respectively]. Women who had undergone RRM were more likely to undergo RRSO, with an odds ratio of 3.66; 95 % CI (1.12–11.98) in multivariable analysis.

No significant increased odds of undergoing a RRSO were identified if there was a first or a second/third degree relative with a history of ovarian cancer [OR 0.61; 95 % CI (0.23–1.62) and OR 1.30; 95 % CI (0.37–4.57) respectively]. This also accounted for women with a first or second/third degree relative with a history of breast cancer [OR 0.85; 95 % CI (0.31–2.33) and OR 1.30; 95 % CI (0.49–3.44) respectively]. Family history of ovarian and/or breast cancer evaluated altogether also showed no significant increased odds for undergoing RRSO [OR 1.10; 95 % CI (0.44–2.76)].

## Discussion

We show that RRSO uptake is high after counseling and that only few consultations at the gynecologist are needed, to decide to undergo RRSO.

Secondly, women with a personal medical history of breast cancer do not seem to be more likely to undergo RRSO, than those without. This also accounts for women with a family history of ovarian and/or breast cancer.

Reported RRSO uptake in the literature varies from 10.9 to 75 % [22–34]. In comparison, RRSO uptake in this study is relatively high. An explanation for this difference is that in most studies, screening for ovarian cancer is offered as an alternative to undergoing RRSO [23, 26–29, 32–34], even though in the meantime it had sufficiently been shown that this was not effective in reducing ovarian cancer mortality [13–19].

It has been published that RRSO uptake is higher in women with a personal medical history of breast cancer [22, 23, 27, 29]. But only two studies could support this with significant results in multivariable analyses [27, 29]. In two other studies, RRSO uptake was even *lower* in women with a medical history of breast cancer [28, 32].

In several studies, RRM in personal medical history also seemed to have an influence, showing positive odds ratios in univariable analyses [7, 22, 23, 28].

Family history of ovarian and/or breast cancer were thought to be factors of influence, as some studies conclude [23, 27, 30, 33], although not all analyses were multivariable with adjustment for known factors of influence [27, 33].

A limitation of this study is that information bias might have occurred because of the study design. Although not all possible factors of influence in the decision making of undergoing RRSO (i.e. demographic factors) could be evaluated, for lack of available information. Detailed information on family history was not present for every patient, but it was collected as thoroughly as possible by standardized questioning by the experts.

Our findings are likely influenced by the method of counseling performed and particularly the fact that screening was not offered. Therefore, results of this study may not be extended towards situations where alternative strategies such as screening are offered. However, our results do indicate that a protocol based solely on RRSO counseling, without the alternative option of screening, is effective in establishing a high uptake of RRSO in women with an increased lifetime risk of ovarian cancer. This protocol can be useful in a variety of health-care systems given that most women who ultimately decide for RRSO needed 3 or less counseling sessions. We consider a multidisciplinary approach by a gynecologic oncologist, clinical geneticist and nurse specialist, specialized in familial cancer care, pivotal in this counseling practice.

Future studies may focus on validating the current results, as well as investigating other possible factors of influence (e.g. psychological), possibly within the context of a qualitative study. Especially the subgroup who needed >3 consultations in the RRSO decision making process is of interest with respect to their considerations. Such an analysis may identify opportunities for improving and individualizing the counseling practice.

In conclusion, the majority (87.2 %) of the women carrying a BRCA mutation or having familial susceptibility to ovarian cancer, who visited our clinic, where screening is not offered, opted for RRSO. This decision is made relatively quick; the majority decided after the first consultation with the gynecologist. This approach is therefore likely to be effective in reducing ovarian cancer related mortality in this high risk population. Personal medical history of (breast) cancer was not found to significantly affect the decision to undergo RRSO, though women who had undergone RRM were more likely to opt for RRSO. Also, patients with 1st degree relatives who had breast cancer needed significantly less consultations to decide for RRSO.
